# Entomological assessment of the transmission following recrudescence of onchocerciasis in the Comoé Valley, Burkina Faso

**DOI:** 10.1186/s13071-019-3290-5

**Published:** 2019-01-15

**Authors:** Lassane Koala, Achille S. Nikièma, Alain B. Paré, François Drabo, Laurent D. Toé, Adrien M. G. Belem, Daniel A. Boakye, Soungalo Traoré, Roch K. Dabiré

**Affiliations:** 1 Ministère de l’Enseignement Supérieur, de la Recherche Scientifique et de l’Innovation, Institut de Recherche en Sciences de la Santé (IRSS), Direction Régionale de l’Ouest, BP 545, Bobo Dioulasso 01, Burkina Faso; 2 Ministère de la Santé, Direction Générale de la Santé, BP 7003, Ouagadougou 01, Burkina Faso; 3 WHO/AFRO/ESPEN Laboratory, Ouagadougou, Burkina Faso; 4 Université Nazi Boni de Bobo-Dioulasso 01, BP 1091, Bobo-Dioulasso, Burkina Faso; 5 BP 2938, Ouagadougou 01, Burkina Faso

**Keywords:** Recrudescence, Onchocerciasis, *Simulium*, Transmission, O-150 PCR, Ivermectin, Burkina Faso

## Abstract

**Background:**

Onchocerciasis, or river blindness, is a dermal filariasis caused by infection with the nematode parasite *Onchocerca volvulus*, transmitted to humans through the bites of blackflies of the genus *Simulium.* Despite the decade-long West African Regional Programme for the Elimination of Onchocerciasis, involving the mass administration of ivermectin to populations in endemic areas, recrudescence has occurred. An example is in the Cascades Region of south-west Burkina Faso where the resumption of transmission had resulted in infection prevalences of up to 70% in some villages. In 2011, a strategy for community-directed distribution of ivermectin (CDTI) was set up to respond to this worrying re-emergence.

Here, we report on a study of *Onchocerca* spp. transmission in the affected area carried out from January to December 2012. Every month, host-seeking adult females of the *S. damnosum* complex were collected at sites on the River Comoé near the four villages (Bodadiougou, Bolibana, Badara Karaboro and Badara Dogossè) that had recorded the highest prevalences in 2010. Collected blackflies were dissected and infective larvae were identified using the O-150 PCR method.

**Results:**

A total of 9114 *S. damnosum* (*s.l.*) adult females were collected, of which 5142 were parous (56.4%) and 78 (1.51%) were infective carrying a total of 137 infective larvae. The annual transmission potential (ATP) was calculated as 0, 30, 255 and 771 infective larvae/man/year in Badara Dogossè, Bolibana, Badara Karaboro and Bodadiougou, respectively. Transmission levels in the latter two are of particular concern as they were higher than 100 infective larvae/person/year, the designated minimum threshold required for elimination of severe pathology, including damage to vision.

**Conclusions:**

These results confirm that recrudescence of onchocerciasis has occurred, and that transmission of *O. volvulus* was active at sites on the Comoé River in the Cascades region in 2012. In accordance with WHO recommendations, CDTI should be continued and the situation in the Cascades region should be closely monitored if further spread of this outbreak is to be avoided.

**Electronic supplementary material:**

The online version of this article (10.1186/s13071-019-3290-5) contains supplementary material, which is available to authorized users.

## Background

Onchocerciasis is a sub-dermal filarial infection, resulting from infection with the nematode *Onchocerca volvulus*. This viviparous filaria releases millions of immature microfilariae which migrate to the skin where, unless picked up by a feeding *Simulium* spp., they eventually die, causing the main pathology of the disease onchocerciasis or river blindness [1]. If ingested during a blood meal by *Simulium* (blackfly) vectors, microfilariae develop within the fly to infective stages in the head (L3H), which are transmitted to humans at the vector’s next blood meal [2]. In West Africa, the immature stages of the main vectors, a number of sibling species within the *Simulium damnosum* complex, are found in turbulent fast-flowing rivers and the adults bite humans at high levels close to these sites [1, 3]. The disease is currently found in 31 countries in sub-Saharan Africa, in Yemen and in the Americas [4, 5]. Prior to the 1970s, onchocerciasis was of major public-health importance in West Africa, but the successes of the Onchocerciasis Control Programme in West Africa (OCP) and its successor, the African Programme for Onchocerciasis Control (APOC), have eliminated or reduced transmission levels in many areas. Initially OCP was a vector control programme, but then was changed to a mass drug administration programme under APOC with the arrival of mectizan (ivermectin). When OCP ceased larviciding in 1989, the transmission of onchocerciasis had been interrupted in the vast area of the programme’s core, including the Comoé basin. When activities ended in 2002, 600,000 cases of blindness had been prevented, 18 million children born inside the area were free from the risk of blindness, and 25 million hectares were now safe for resettlement [2]. Responsibility for maintaining the OCP’s achievements was devolved to national ministries of health, who were to operate entomological and epidemiological surveillance and response programmes and strategies, to prevent any recrudescence of the disease.

In 2010 and 2011, surveillance by the Programme National de Lutte contre l’Onchocercose (PNLO) in Burkina Faso discovered worrying levels of *O. volvulus* infection in villages of the Cascades Region [6]. Of 28 villages evaluated along the River Comoé, 22 had prevalence rates ranging from 0.67 to 70.9%, 13 of which exceeded the tolerable threshold of 5%. To control this recrudescence, the PNLO and its partners (Sightsavers and WHO/APOC) introduced community-directed treatment with ivermectin (CDTI) twice per year in the affected districts [6].

The origin of this recrudescence is a key unanswered question, although the migration of infective blackflies one of the most likely hypotheses, given that the Cascades region of Burkina Faso shares a border with Côte d'Ivoire, a country where some areas are still endemic for onchocerciasis [7]. Reinvasion of infected vectors from outside controlled zones is a major threat to the success of onchocerciasis control programmes in Africa [8].

The high prevalence rates recorded in the surveillance operation were alarming, and it was decided that investigations into transmission in the area should begin. In June and July 2011, preliminary entomological work carried out in Bolibana and Badara Karaboro (the villages where prevalences of 46.25 and 70.97%, respectively, had been recorded that year) led to estimates for the monthly transmission potential (MTP) of up to 469 and 351 L3H/person/month at the respective sites [9]. These MTPs were higher than the tolerable threshold for the annual transmission potential (ATP) of 100 L3H/person/year, below which onchocerciasis would not be of a public health significance [10]. Based on these findings, a recommendation was made to the PNLO to collect data on blackfly biting and infection rates in order to determine the ATP and to provide a baseline for future monitoring and evaluation. The results of that study are reported here. The following points were considered: (i) annual variations in the populations of blackflies; and (ii) changes in the mean age of the vector and its potential of transmission. This publication reports the results of the longitudinal entomological survey conducted on the Comoé basin in the Cascades Health Region.

## Methods

### Description of the study area

**Fig. 1:**
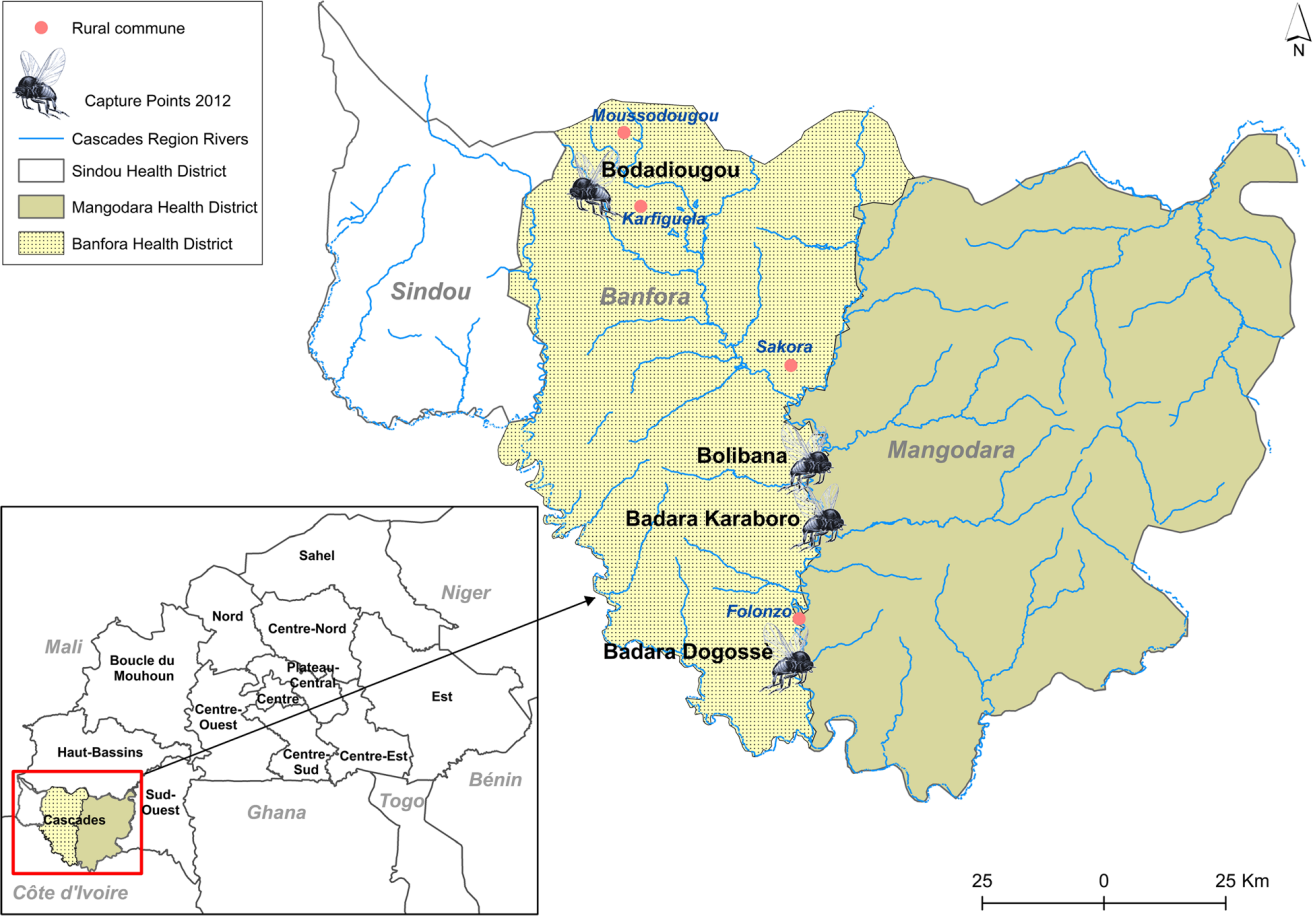
Location of the capture points in the Cascades Region (Burkina Faso)

The study was carried out from January to December 2012 in the Comoé River basin, which crosses the entire Cascades region located to the southwest of Burkina Faso (10°40'N, 4°50'W; elevation 425 m above sea level). This region is one of 13 administrative regions of the country. Historically, it was within the original OCP area because of the presence of two rivers, the Comoé and Léraba, with several *Simulium damnsoum* breeding sites [1, 11]. The region has a southern Sudannian climate, described by two major seasons: the wet season from April to October and the dry season from November to March. Precipitation ranges from 1000 to 1200 mm of rain per year and the average annual temperatures are between 17–36 °C. The Cascades region (Fig. 1) has 3 health districts (Banfora, Mangodara and Sindou) and 75 Health and Social Promotion Centers (CSPS). Since 2011, 21 CSPS within the Banfora and Mangodara health districts, including those covering the villages in our study area, have applied ivermectin through CDTI twice-yearly at 6-month intervals. In the study area *S. damnosum* (*s.s.*) was the major vector during the dry season while *S. sirbanum* was more common in the rainy season [12, 13].

### Choice of capture points

Following the resurgence of onchocerciasis in the Cascades region in 2010, we selected four villages from those with the highest prevalence; all were in the Comoé basin, near the villages of Bodadiougou, Badara Karaboro, Bolibana and Badara Dogossè [6]. The choice of capture points was made to ensure it was located in the shade, sheltered from the wind and usually accessible all year round [1, 14].

### Bodadiougou capture point

**Fig. 2:**
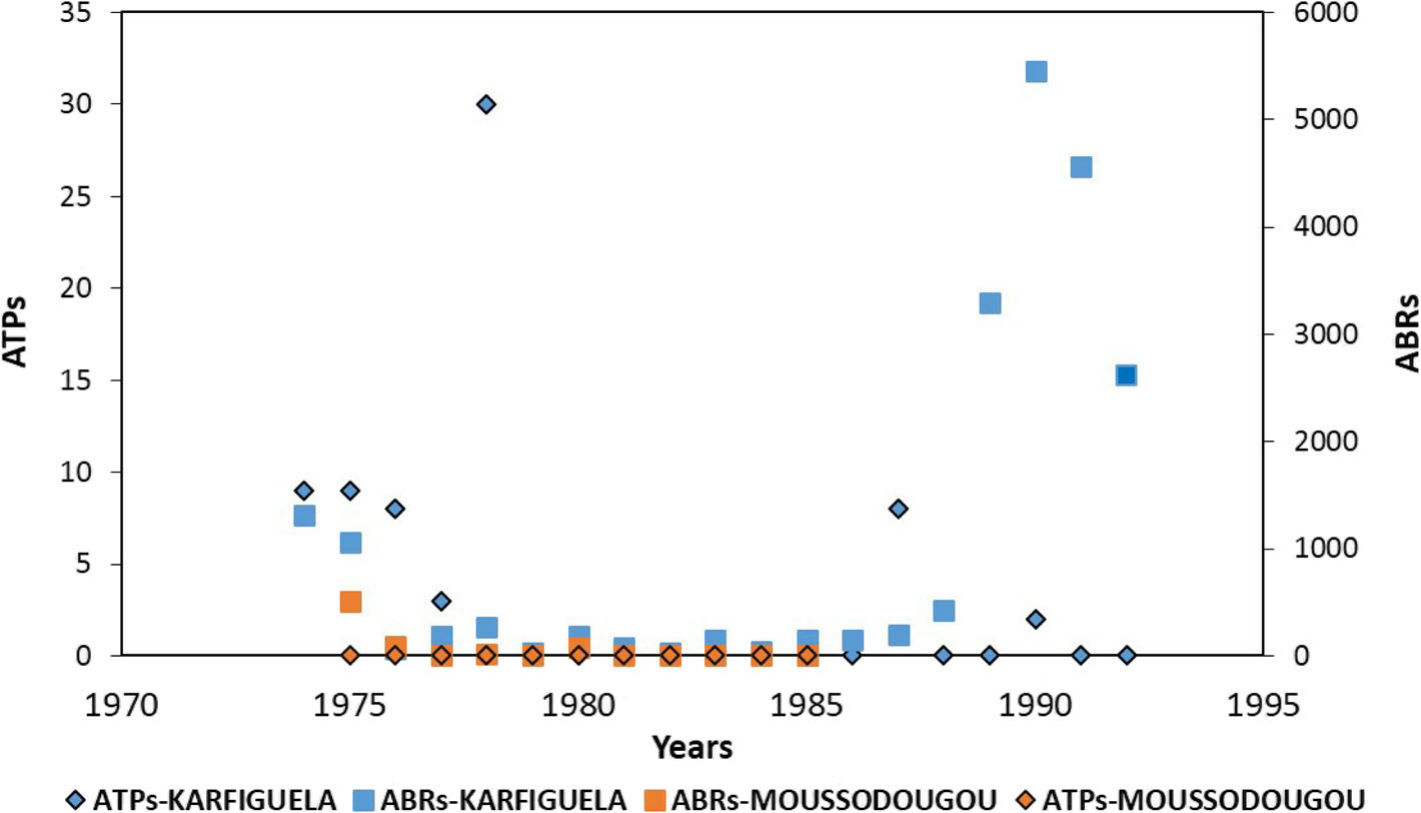
Entomological data of the Onchocerciasis Control Programme in West Africa from 1975–1985 for Moussodougou and 1974–1992 for the village of Karfiguela. Larviciding was stopped in 1989. *Abbreviations:* ATP, annual transmission potential; ABR, annual biting rate

The village of Bodadiougou is located about 12 km from the rural commune of Moussodougou (see Fig. 1). The village is under the CSPS of Toumousseni and had a population of 1746 people in 2010. The prevalence of onchocerciasis in this village was 5.72% in 2010 [6]. Historically, the OCP carried out larviciding (from 1974 to 1989) in the breeding sites of Bodadiougou and in those of the neighboring villages, Moussodougou and Karfiguela. Figure 2 summarizes the results of the OCP’s vector control activities at Moussodougou and Karfiguela. The village of Bodadiougou is characterized by the presence of two dams, the Lobi Dam created in 1975 and the Comoé Dam created in 1991.

### Badara Karaboro and Bolibana capture points

Both villages are located about 15 km from the rural commune of Nofesso. Bolibana and Badara Karaboro are covered by the Nofesso CSPS with populations of 357 and 90, respectively, in 2010. The prevalence of onchocerciasis was 46.25% at Bolibana and and 70.97% at Badara Karaboro. There are no historical entomological data from the OCP in the region, but the nearest OCP sentinel village is Sakora village, which is also covered by the Nofesso CSPS, was the subject of several epidemiological evaluations by the OCP [6].

### Badara Dogossè capture point

**Fig. 3:**
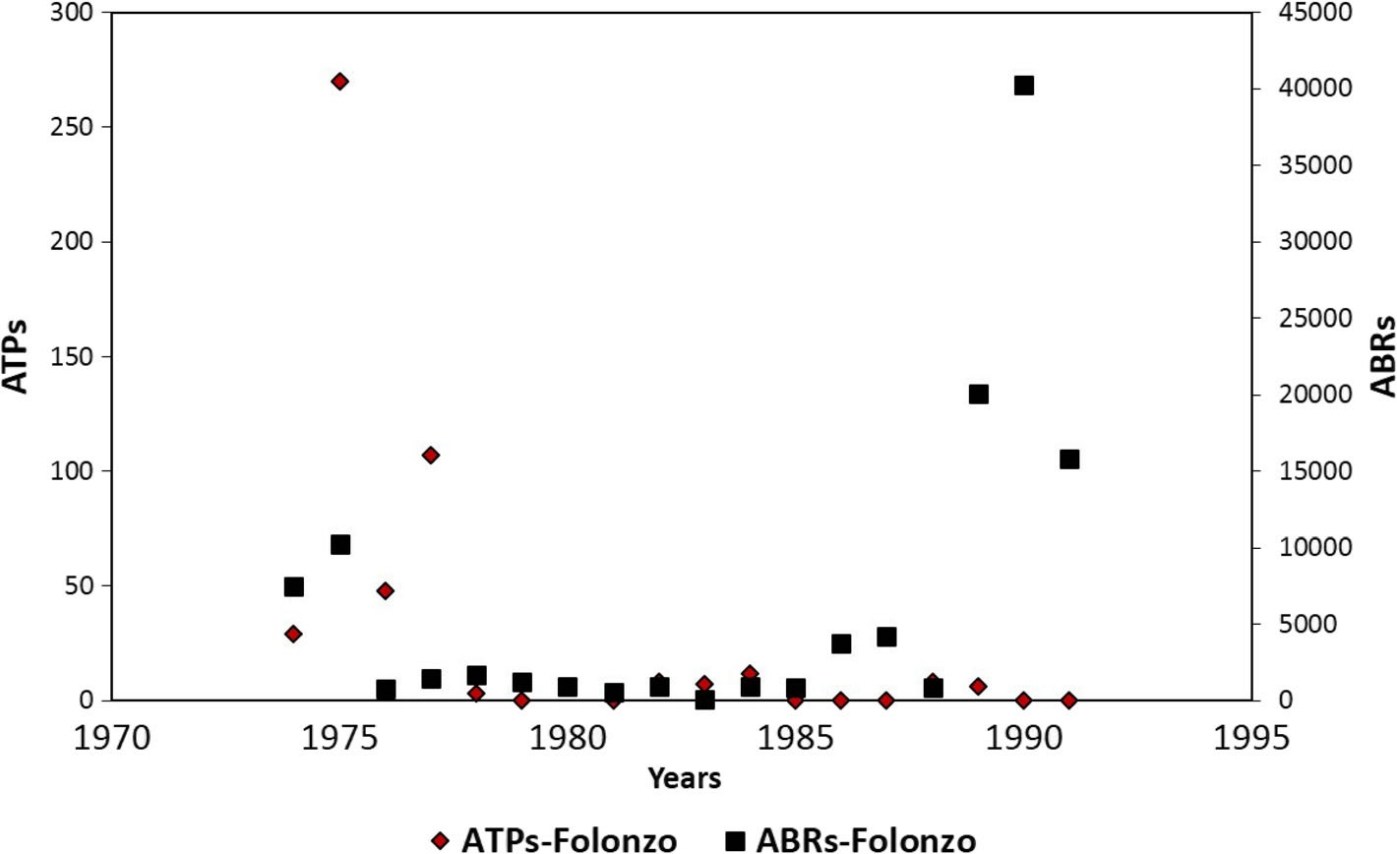
Entomological data of the Onchocerciasis Control Programme in West Africa from 1975–1985 at blackfly breeding sites in Folonzo. *Abbreviations:* ATP, annual transmission potential; ABR, annual biting rate

The village of Badara Dogossè is located about 10 km from the rural town of Folonzo. Badara Dogossè is under the umbrella of the Folonzo CSPS with a population of 152 in 2010. The prevalence of onchocerciasis in this village was 13% in 2010 [6]. Historically, the OCP programme regularly carried out larviciding and entomological monitoring activities from 1974 to 1985 at the breeding sites of Folonzo. Figure 3 summarizes the entomological results from OCP at Folonzo breeding sites from 1974 to 1991. Notably, the Folonzo region has been subject to reinvasion by migrating blackflies [6].

### Human landing collection (HLC)

Adult *Simulium* spp. were collected by human landing catches (HLC) [1, 14] by experienced field workers. After collection, tubes were packed in batches of five in cotton soaked with water to keep the flies alive. The HLCs were made from 7:00 h to 18:00 h on two consecutive days monthly, from January to December 2012.

### Identification of blackflies

The morphological identification of blackflies was done in two consecutive steps. First, *Simulium damnosum* (*s.l.*) were distinguished from other species according to the criteria described in Davies & Crosskey [15]. Secondly, the coloration of the wing tufts, procoxa, antennae and scutal tufts were used to distinguish the savanna cytospecies [*S. damnosum* (*s.s.*) and *S. sirbanum*] from the other cytospecies [15, 16].

### Dissection of blackflies and preservation of infected larvae

The dissection of blackflies was carried out as described previously [15]. Briefly, the abdomens of individual females were opened in a drop of physiological solution (normal saline) and ovaries examined to determine their physiological age: nulliparous flies were not processed further; tissues of parous flies were teased apart in a drop of physiological solution (normal saline) and then thoroughly examined for filarial parasites morphologically indistinguishable from *O. volvulus* larvae [17]. When a fly was determined to be infective, the infective larvae present in its head (L3H) were harvested and preserved, with part of its Malpighian tubules between a slide and coverslip as previously described [18]. These slides were sent to the molecular biology laboratory for identification to species level.

### Molecular analysis

Infected larvae were identified to species level by molecular analyses at the Multi-diseases Surveillance Center of APOC (current ESPEN Labs).The larvae were amplified first by the O-150 PCR amplification technique and then the PCR products were hybridized to the OVS-2 oligonucleotide probes specific to *Onchocerca volvulus* [19, 20]. This allowed *Onchocerca* species of animal and human origins to be distinguished and allowed more accurate measures of the entomological indicators of transmission.

### Entomological indices

The original protocol, used to evaluate the effect of control on onchocerciasis transmission in the OCP, was also used here [21]. This protocol employs several entomological indicators to estimate onchocerciasis transmission: the parous rate, biting rate and transmission potential.

The parous rate is the proportion of dissected flies that were classed as parous, at each capture point.

The biting rate represents the number of bites received by an exposed person in any given period. We calculated the monthly biting rate (MBR) of each site as the total number of blackflies collected in the month divided by the number of days of capture in this month multiplied by 30. The annual biting rate (ABR), which was the main biting rate indicator, was calculated as the arithmetic sum of the MBRs for 12 months.

In the savanna areas of West Africa, the WHO considered entomological results as satisfactory when the ABR was less than 1000 bites/person/year [21].

### Transmission potential

We calculated the monthly transmission potential (MTP) as the MBR of the month multiplied by the total number of L3H for that month divided by the total number of flies dissected. The annual transmission potential (ATP) was calculated as the arithmetic sum of the MTP of twelve months (January to December).

In the savanna areas of West Africa, the WHO/OCP considers entomological results as satisfactory when the ATP was less than 100 L3H/person/year [21] and recently, the WHO (2016) stated that an annual rate of 20 L3H/person/year is a suitable cut-off point for onchocerciasis elimination [3].

### Infectivity rates and parasitic loads

We calculated the infectivity rate as the total number of infective flies with larva in the head (FLH) multiplied by 1000 and divided by the total number of parous flies. The WHO stipulated that entomological results could be considered satisfactory when the infectivity rate was less than 1 infective blackfly per 1000 parous flies. This threshold below which the infectivity rate could be considered satisfactory was determined by the using of the ONCHOSIM model [22, 23]. The parasite load of dissected *Simulium* spp. was also calculated by multiplying the number of infective larvae (L3H) by 1000, then divided by the total number of parous flies.

## Results

### *Simulium* spp. relative abundance and biting rates

From January to December 2012, a total of 94 days was spent sampling, at a rate of 2 days/month at all villages except Bodadiougou and Badara Dogossè where, due to unusually heavy rain, only 1 day of capture was performed in December.

The results obtained in each village are summarized in Additional file 1: Tables S1-S4 for Bodadiougou, Bolibana, Badara Karaboro, Badara Dogossè, respectively. A total of 9114 females of blackflies were captured and morphologically identified as savanna species: *S. damnosum* (*s.s.*) and *S. sirbanum*. The peak biting rates were recorded during the rainy season, May to October, with 231, 227 and 209 bites/human/day recorded in Bolibana, Badara Karaboro and Badara Dogossè, respectively. Conversely, in Bodadiougou, the highest densities were recorded during the dry season (February to April) with a peak in February (1432 bites/human/day). The ABR were calculated as 12,750 in Badara Dogossè, 22,335 in Bolibana, 22,365 in Badara Karaboro and 84,495 bites/ man/year in the village of Bodadiougou.

### Parous rates

The mean parous rate of all *Simulium* spp. in the study was 74.81%. Although there was no difference in the mean parous rates between the two seasons in the villages (73.76% during the dry season *vs* 70.31% during the rainy season), rates ranged between 43.33–100% in Badara Dogossè, 55.56–90.82% in Badara Karaboro and 25.00–95.70% in Bolibana. The mean parous rates recorded during the 7 months of the rainy season reached 83.49% in Badara Karaboro, 80.35% in Bolibana and 73.37% in Badara Dogossè. Rates were lower during the dry season, averaging 49.91, 44.08 and 44.93% in Badara Karaboro, Bolibana and Badara Dogossè, respectively. No Simuliidae were caught during the month of March, from the breeding sites of Badara Karaboro and Badara Dogossè. In Bodiadougou, parous rates fluctuated from 31.36 to 97.37% without any distinct difference between seasons: the mean parous rate was 71.28% for the rainy season and 73.09% for the dry season.

### Raw and corrected indicators of transmission

#### Onchocerca volvulus infectivity rate and parasite loads in Simulium spp.

The transmission indicators (infectivity rates, parasite loads and ATPs) were corrected to ensure that they represented infections with *O. volvulus* only. A total of 6884 out of the 9114 female blackflies captured were dissected of which 5142 were parous, 175 were infected, and 78 were carrying a total of 137 infective larvae (L3H). All 137 infective larvae (Bodadiougou, *n* = 93; Bolibana, *n* = 21; Badara Karaboro, *n* = 23) collected during the dissections were identified using molecular methods (see Additional file 1: Table S5) and 52 were identified as *O. volvulus*: 33/93 from Bodadiougou, 2/21 from Bolibana and 17/23 from Badara Karaboro. The remaining 85 were identified as other *Onchocerca* species.

The raw infectivity rates (no. of FLH/1000 parous flies) for the different sites were 0 in Badara Dogossè, 12 in Bolibana, 14 in Badara Karaboro and 22 in Bodiadougou. After corrections, these rates become 11 in Badara Karaboro, 2 in Bolibana and 7 FLH/1000 parous flies at Bodadiougou (see Additional file 1: Table S5). The parasite load values were adjusted from raw values of 19, 20, and 41 to corrected values of 2, 4 and 14 L3H/1000 parous flies in Bolibana, Badara Karaboro and Bodiadougou, respectively. In Badara Dogossè, stage I and II larvae were harvested from sampled blackflies, but no L3 were found.

#### Annual transmission potential (ATP)

The results of the various entomological indicators before and after molecular analyses are summarized in Table 1. Transmission was higher in the rainy season in Bolibana and Badara Karaboro while the reverse was observed in Bodiadougou. The highest MTP (645 infective larvae/human/month) was recorded in February in the village of Bodadiougou and the lowest MTP (0 infective larvae/human/year) were recorded in March in both Badara Karaboro and Badara Dogossè. The raw ATP was 0 in Badara Dogossè, 315 in Bolibana, 345 in Badara Karaboro and 2385 in Bodiadougou. After correction, *O.volvulus* ATPs were determined to be 30, 255 and 771 at Bolibana, Badara Karaboro and Bodadiougou, respectively.

**Table 1:** Raw and corrected values of entomological indicators of onchocerciasis transmission along the Comoé River

Sites	ABR (bites/man/year)	Parity rate (%)	Infective female/1000 parous flies		Infective larvae/1000 parous flies		ATPs (infective larvae/man/year)	
			Gross	Corrected	Gross	Corrected	Gross	Corrected
Bodadiougou	84,495	72,92	22	7	41	14	2385	771
Badara Karaboro	22,335	72,67	14	11	20	4	345	255
Bolibana	22,365	78,74	12	2	19	2	315	30
Badara Dogossè	12,750	73,92	0	0	0	0	0	0

## Discussion

The Comoé River, a perennial fast flowing river with numerous *S. damnosum* breeding sites, has long been recognized as one of the most important sources of onchocerciasis vectors in this region [1]*.* The results reported here raise the question of whether the new infections detected in the local human population indicate that the Comoé’s blackflies are responsible for a resurgence of the disease or whether the infective vectors originated elsewhere. With the exception of Bodadiougou, adult blackfly densities were higher during the rainy season than during the dry season and the lowest vector densities occurred between February and April at all sites except Bodadiougou. In fact, no blackflies were collected in March in Badara Karaboro and Badara Dogossè because the Comoé had dried up completely at these points. Densities typically start to increase in May when the rains begin, and biting rates can exceed 100 bites/person/day in each of the three villages during the rainy season. Indeed, the biting rates and patterns in these sites were described previously by Philippon & LeBerre [1, 14] to illustrate the blackfly burden and the risk of onchocerciasis transmission to which populations in West Africa were exposed. At the height of the rainy season, the rise in the river Comoé’s level submerges the rocks and vegetation where the preimaginal stages of *S. damnosum* would otherwise be attached, thus reducing significantly the size of blackfly populations emerging from this breeding site. While local production of blackflies might be maintained by the Comoé river’s tributaries, the physiological ages of the flies collected during the rainy season suggest otherwise.

In this study, the 74.88% parous rate recorded during the rainy season was markedly higher than the 54.3% recorded during the dry season, and the inverse of the norm where wet season parous rates are low because of the extremely high proportions of newly emerged individuals in populations during this season [14]. Hence, the combination of low emergence rates of local blackflies and high biting rates of parous adult females led us to hypothesize that the Comoé basin area around Badara Dogossè, Bolibana and Badara Karaboro may be subject to invasion by migrating blackflies during the rainy season. This suggestion is corroborated by previous studies carried out by the OCP, when the invasion of rivers systems in Côte d'Ivoire, Burkina Faso and Mali (including the River Comoé) by waves of *S. sirbanum* and *S. damnosum* (*s.s.*) with high parous rates occurred early in the rainy season [11, 24]. In Bodadiougou, the situation may differ. Here, our results indicated that blackflies were present throughout 2012, contrary to what the OCP historical data show. Bodadiougou is situated close to two large dams, the “dam Comoé” (also known as “Barrage de Moussodougou”) and the “dam Lobi”, both located upstream of the breeding sites. The construction of these dams created optimal conditions for blackflies, especially when sluices are opened during dry periods. The biting rate/person/day reached a peak of 716 in February in our study, leading to the significant nuisance caused by these “off-season” flies to the population of Bodadiougou where they are also responsible for dry season maintenance of transmission.

The molecular identification of the 137 infective larvae allowed the separation of *O. volvulus* (38%) from other species of *Onchocerca* (62%). The high proportion of non-human *Onchocerca* species can be explained by the fact that many *Simulium* spp. feed on animals as well as humans and can also transmit species of *Onchocerca* of animal origin such as *O. ochengi*, a common parasite of cattle in West Africa with L3H resembling those of *O. volvulus* [25]*.* In addition, it is known that *Simulium sirbanum* is a species with marked zoophily [14, 26]. The study area is an area of transhumance and provides grazing for livestock supporting breeders from the Peulh ethnic group with large cattle herds. Even after accounting for the presence of animal-derived *Onchocerca* parasites, the results indicate significant ongoing onchocerciasis transmission, particularly in the villages of Bodadiougou and Badara Karaboro.

**Fig. 4:**
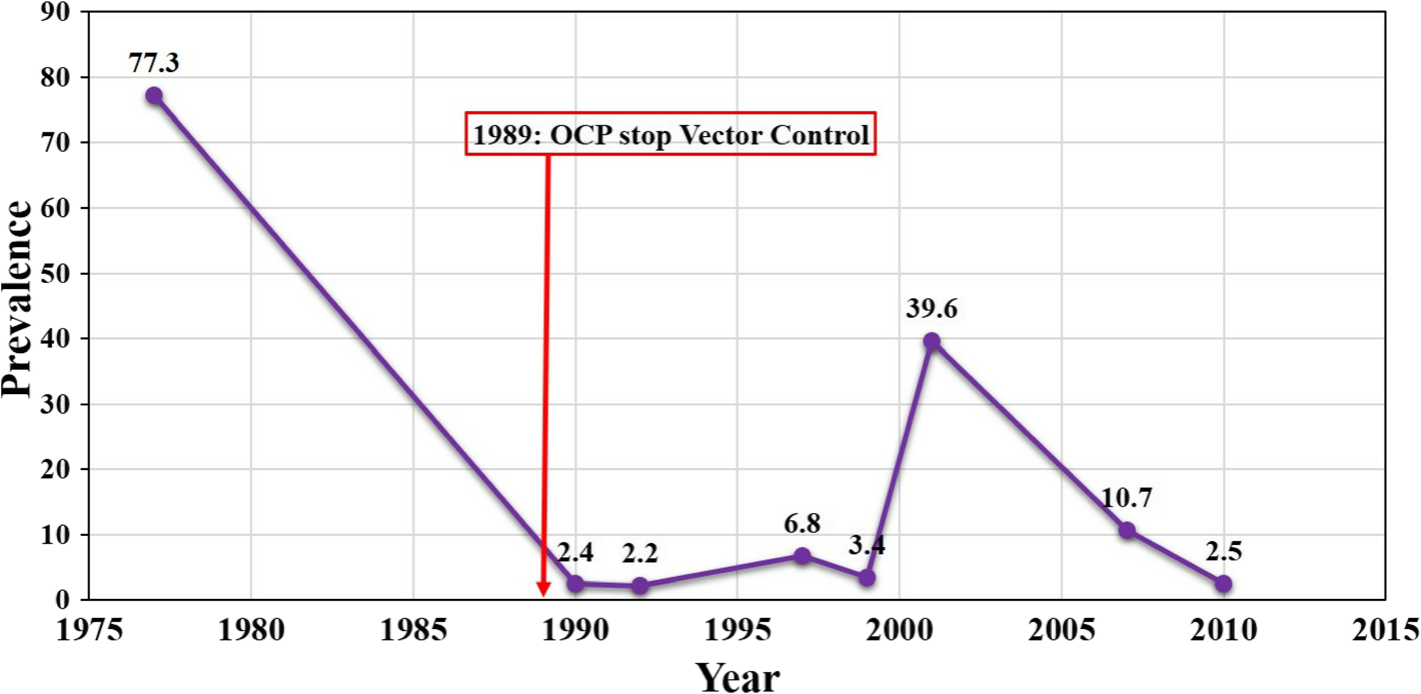
Evolution of onchocerciasis prevalence at Sakora village from 1977 to 2010. *Abbreviation*: OCP, Onchocerciasis Control Programme

In the framework of elimination, the WHO [3] stipulates that entomological results are satisfactory when the vector infectivity rate is less than one infective blackfly/1000 parous flies representing a prevalence of 0.1%. Our results show infectivity rates of 2, 7 and 11 FLH/1000 parous flies in Bolibana, Bodadiougou and Badara Karaboro, respectively, exceeding the WHO threshold at all sites except Badara Dogossè. The highest corrected ATPs were recorded in Bodadiougou (771 L3H /man/year) and Badara Karaboro (255 L3H/man/year). At Badara Dogossè, no infective larvae were found but the presence of developing larvae indicated the presence of residual infections. However, the WHO [3] stipulates that an ATP of 20 L3/man/year is the threshold in the framework of elimination of onchocerciasis, and Bodadiougou, Badara Karaboro and Bolibana exceeded this threshold. One possible explanation for this recrudescence is the existence of residual foci of disease transmission at the end of OCP. In 2001, there was a recrudescence of onchocerciasis at Sakora village (prevalence of 39%) along the Comoé River in the region of Folonzo [6] (see Fig. 4). The OCP formally ceased its operations in December 2002 and the responsibility for onchocerciasis surveillance and any necessary control operations such as ivermectin treatment in the sentinel sites (including Sakora village) was devolved to the Ministry of Health. The Comoé basin in Burkina Faso was never subjected to ivermectin for onchocerciasis treatment, but mass drug administration against lymphatic filariasis was initiated by the Ministry of Health in 2004. The prevalence in Sakora village dropped to 10.3% in 2007 and to 4.2% in 2010 [6] but the possibility that this focus was a source of the infection for villages nearby, such as Bolibana and Badara Karaboro, cannot be excluded.

## Conclusions

These results confirm that recrudescence of onchocerciasis has occurred, and that transmission of *O. volvulus* was active at sites on the Comoé River in the Cascades region in 2012. In accordance with WHO recommendations, CDTI should be continued and the situation in the Cascades region should be closely monitored if higher levels or the spread of onchocerciasis are to be avoided [9].

## Additional file

Additional file 1: **Table S1.** Bodadjougou’s onchocerciasis entomological indicators of transmission. **Table S2.** Bolibana onchocerciasis entomological indicators of transmission **Table S3.** Badara Karaboro onchocerciasis entomological indicators of transmission. **Table S4.** Badara Dogossè de Folonzo onchocerciasis entomological indicators of transmission **Table S5.** Molecular identification of the infected larvae harvested. (DOCX 30 kb)

## Abbreviations

ABR: Annual biting rate
APOC: African Programme for Onchocerciasis Control
ATP: Annual transmission potential
CDTI: Community-directed treatment with ivermectin
CSPS: Centre de Santé de Promotion Social
ESPEN: Expanded Special Project for Elimination of Neglected tropical diseases
FLH: Female with larvae in head
HLC: Human landing collection
L3H: Infective larvae in head
MBR: Monthly biting rate
MTP: Monthly transmission potential
NGO: Non-governmental organization
OCP: Onchocerciasis Control Programme in West Africa
PCR: Polymerase chain reaction
PNLO: Programme National de Lutte contre l’Onchocercose
WHO: World Health Organization
