# Supplementary material for: Entomological assessment of the transmission following recrudescence of onchocerciasis in the Comoé Valley, Burkina Faso
Source: Parasit Vectors. 2019 Jan 15;12:34. doi: 10.1186/s13071-019-3290-5 (PMC6332526; doi:10.1186/s13071-019-3290-5)
Supplement: Supplementary file 1 — Table S1. Bodadjougou’s onchocerciasis entomological indicators of transmission. Table S2. Bolibana onchocerciasis entomological indicators of transmission Table S3. Badara Karaboro onchocerciasis entomological indicators of transmission. Table S4. Badara Dogossè de Folonzo onchocerciasis entomological indicators of transmission Table S5. Molecular identification of the infected larvae harvested. (DOCX 30 kb) [file 13071_2019_3290_MOESM1_ESM.docx]

**Additional file 1**

**Table S1.** Bodadiougou’s onchocerciasis entomological indicators

| Indicators |  | | | | | | | | | | | | |
| --- | --- | --- | --- | --- | --- | --- | --- | --- | --- | --- | --- | --- | --- |
| Months | Jan | Feb | Mar | Apr | May | June | July | Aug | Sept | Oct | Nov | Dec |  |
| No of days | 2 | 2 | 2 | 2 | 2 | 2 | 2 | 2 | 2 | 2 | 2 | 1 |  |
| No of flies collected | 304 | 1432 | 754 | 675 | 542 | 503 | 75 | 83 | 204 | 136 | 297 | 314 |  |
| No of bites/man/day | 152 | 716 | 377 | 338 | 271 | 251.5 | 38 | 41.5 | 102 | 68 | 149 | 314 |  |
| MBR | 4560 | 21480 | 11310 | 10125 | 8130 | 7545 | 1125 | 1245 | 3060 | 2040 | 4455 | 9420 |  |
| No of flies dissected | 304 | 433 | 414 | 424 | 339 | 357 | 75 | 83 | 204 | 136 | 140 | 182 |  |
| No of parous flies | 296 | 385 | 318 | 327 | 226 | 218 | 58 | 64 | 132 | 102 | 91 | 68 |  |
| % parous flies | 97.37 | 88.91 | 76.81 | 77.12 | 66.67 | 61.06 | 77.33 | 77.11 | 64.71 | 75 | 65 | 37.36 |  |
| No of infected flies | 13 | 12 | 25 | 16 | 12 | 9 | 0 | 3 | 6 | 5 | 0 | 3 |  |
| % infected flies | 4.39 | 3.12 | 7.86 | 4.89 | 5.31 | 4.13 | 0 | 4.69 | 4.55 | 4.9 | 0 | 4.41 |  |
| No of infective flies | 7 | 9 | 7 | 6 | 9 | 3 | 0 | 1 | 5 | 2 | 0 | 1 |  |
| % infective flies | 2.36 | 2.34 | 2.2 | 1.83 | 3.98 | 1.38 | 0 | 1.56 | 3.79 | 1.96 | 0 | 1.47 |  |
| No of Infective flies/1000pares | 24 | 23 | 22 | 18 | 40 | 14 | 0 | 16 | 38 | 20 | 0 | 15 |  |
| No of infective larvae (L3H) | 11 | 13 | 20 | 11 | 13 | 7 | 0 | 1 | 8 | 8 | 0 | 1 |  |
| No of L3H/1000 parous | 37 | 34 | 63 | 34 | 58 | 32 | 0 | 16 | 61 | 78 | 0 | 15 |  |
| MTP | 165 | 645 | 546 | 263 | 312 | 148 | 0 | 15 | 120 | 120 | 0 | 52 |  |

**Table S2.** Bolibana onchocerciasis entomological indicators of transmission

| **Indicators** |  |  |  |  |  |  |  |  |  |  |  |  |
| --- | --- | --- | --- | --- | --- | --- | --- | --- | --- | --- | --- | --- |
| Months | Jan | Feb | Mar | Apr | May | Jun | Jul | Aug | Sept | Oct | Nov | Dec |
| No of days | 2 | 2 | 2 | 2 | 2 | 2 | 2 | 2 | 2 | 2 | 2 | 2 |
| No of flies collected | 195 | 8 | 4 | 93 | 159 | 121 | 211 | 231 | 17 | 126 | 150 | 174 |
| No of bitting /man/day | 98 | 4 | 2 | 47 | 80 | 61 | 106 | 116 | 9 | 63 | 75 | 87 |
| MBR | 2925 | 120 | 60 | 1395 | 2385 | 1815 | 3165 | 3465 | 255 | 1890 | 2250 | 2610 |
| No of flies dissected | 195 | 8 | 4 | 93 | 159 | 121 | 211 | 231 | 17 | 126 | 150 | 174 |
| No of parous flies | 125 | 3 | 1 | 89 | 128 | 87 | 177 | 192 | 9 | 119 | 70 | 82 |
| % parous flies | 64.1 | 37.5 | 25 | 95.7 | 80.5 | 71.9 | 83.89 | 83.12 | 52.94 | 94.44 | 46.67 | 47.13 |
| No of infected flies | 3 | 0 | 0 | 0 | 2 | 4 | 10 | 14 | 0 | 0 | 1 | 1 |
| % fem infected | 2.4 | 0 | 0 | 0 | 1.56 | 4.6 | 5.65 | 7.29 | 0 | 0 | 1.43 | 1.22 |
| No of infective flies | 2 | 0 | 0 | 0 | 1 | 2 | 3 | 4 | 0 | 0 | 0 | 0 |
| % infective flies | 1.6 | 0 | 0 | 0 | 0.78 | 2.3 | 1.69 | 2.08 | 0 | 0 | 0 | 0 |
| No of Infective flies/1000parous | 16 | 0 | 0 | 0 | 8 | 23 | 17 | 21 | 0 | 0 | 0 | 0 |
| No of infective larvae (L3H) | 4 | 0 | 0 | 0 | 1 | 2 | 10 | 4 | 0 | 0 | 0 | 0 |
| No of L3H/1000 parous | 32 | 0 | 0 | 0 | 8 | 23 | 56 | 21 | 0 | 0 | 0 | 0 |
| MTP | 60 | 0 | 0 | 0 | 15 | 30 | 150 | 60 | 0 | 0 | 0 | 0 |

**Table S3.** Badara Karaboro onchocerciasis entomological indicators of transmission

| **Indicators** |  | | | | | | | | | | | | |
| --- | --- | --- | --- | --- | --- | --- | --- | --- | --- | --- | --- | --- | --- |
| Sites | Jan | Feb | Mar | Apr | May | Jun | Jul | Aug | Sept | Oct | Nov | Dec |  |
| No of days | 2 | 2 | 2 | 2 | 2 | 2 | 2 | 2 | 2 | 2 | 2 | 2 |  |
| No of flies collected | 172 | 7 | 0 | 98 | 202 | 223 | 227 | 155 | 73 | 67 | 84 | 183 |  |
| No of bites/man/day | 86 | 4 | 0 | 49 | 101 | 112 | 114 | 78 | 37 | 34 | 42 | 92 |  |
| MBR | 2580 | 105 | 0 | 1470 | 3030 | 3345 | 3405 | 2325 | 1095 | 1005 | 1260 | 2745 |  |
| No of flies dissected | 172 | 7 | 0 | 98 | 202 | 223 | 227 | 155 | 73 | 67 | 84 | 183 |  |
| No of parous flies | 156 | 3 | 0 | 89 | 174 | 203 | 160 | 111 | 63 | 59 | 47 | 109 |  |
| % parous flies | 90.7 | 42.86 | 0 | 90.82 | 86.14 | 91.03 | 70.48 | 71.61 | 86.3 | 88.06 | 55.95 | 59.56 |  |
| No of infected flies | 3 | 0 | 0 | 0 | 4 | 11 | 2 | 3 | 1 | 2 | 2 | 1 |  |
| % infected flies | 1.92 | 0 | 0 | 0 | 2.3 | 5.42 | 1.25 | 2.7 | 1.59 | 3.39 | 4.26 | 0.92 |  |
| No of infective flies | 2 | 0 | 0 | 0 | 2 | 6 | 1 | 1 | 1 | 2 | 0 | 1 |  |
| % infective flies | 1.28 | 0 | 0 | 0 | 1.15 | 2.96 | 0.63 | 0.9 | 1.59 | 3.39 | 0 | 0.92 |  |
| No of Infective flies/1000parous | 13 | 0 | 0 | 0 | 11 | 30 | 6 | 9 | 16 | 34 | 0 | 9 |  |
| No of Infective larvae (L3H) | 2 | 0 | 0 | 0 | 2 | 8 | 1 | 1 | 4 | 3 | 0 | 2 |  |
| No of L3H/1000 parous | 13 | 0 | 0 | 0 | 11 | 39 | 6 | 9 | 63 | 51 | 0 | 18 |  |
| MTP | 30 | 0 | 0 | 0 | 30 | 120 | 15 | 15 | 60 | 45 | 0 | 30 |  |

**Table S4**. Badara Dogossè de Folonzo onchocerciasis entomological indicators of transmission

| **Indicators** |  | | | | | | | | | | | | |
| --- | --- | --- | --- | --- | --- | --- | --- | --- | --- | --- | --- | --- | --- |
| Months | Jan | Feb | March | April | May | June | July | Aug | Sept | Oct | Nov | Dec |  |
| No of days | 2 | 2 | 2 | 2 | 2 | 2 | 2 | 2 | 2 | 2 | 2 | 1 |  |
| No of flies collected | 102 | 2 | 0 | 12 | 30 | 42 | 209 | 209 | 36 | 48 | 90 | 35 |  |
| No of bites/man/days | 51 | 1 | 0 | 6 | 15 | 21 | 105 | 105 | 18 | 24 | 45 | 35 |  |
| MBR | 1530 | 30 | 0 | 180 | 450 | 630 | 3135 | 3135 | 540 | 720 | 1350 | 1050 |  |
| No of flies dissected | 102 | 2 | 0 | 12 | 30 | 40 | 209 | 209 | 36 | 48 | 90 | 35 |  |
| No of parous flies | 81 | 1 | 0 | 12 | 13 | 28 | 171 | 171 | 32 | 33 | 42 | 17 |  |
| % parous flies | 79.41 | 50 | 0 | 100 | 43.33 | 70 | 81.82 | 81.82 | 88.89 | 68.75 | 46.67 | 48.57 |  |
| No of infected flies | 1 | 0 | 0 | 0 | 0 | 1 | 2 | 2 | 0 | 1 | 0 | 0 |  |
| % infected flies | 1.23 | 0 | 0 | 0 | 0 | 3.57 | 1.17 | 1.17 | 0 | 3.03 | 0 | 0 |  |
| No of infective flies | 0 | 0 | 0 | 0 | 0 | 0 | 0 | 0 | 0 | 0 | 0 | 0 |  |
| % infective flies | 0 | 0 | 0 | 0 | 0 | 0 | 0 | 0 | 0 | 0 | 0 | 0 |  |
| Nb infective flies/1000parous | 0 | 0 | 0 | 0 | 0 | 0 | 0 | 0 | 0 | 0 | 0 | 0 |  |
| NB infective larvae (L3H) | 0 | 0 | 0 | 0 | 0 | 0 | 0 | 0 | 0 | 0 | 0 | 0 |  |
| NB L3H/1000 parous | 0 | 0 | 0 | 0 | 0 | 0 | 0 | 0 | 0 | 0 | 0 | 0 |  |
| MTP | 0 | 0 | 0 | 0 | 0 | 0 | 0 | 0 | 0 | 0 | 0 | 0 |  |

**Table S5.** Molecular identification of infected larva harvested

| **Lab No.** | **Capture point** | **Collection date** | **No of L3H** | **Identification** |
| --- | --- | --- | --- | --- |
| 34538 | BADARA KARABORO | 24/01/2012 | 1 | *Onchocerca volvulus* |
| 34539 | BADARA KARABORO | 24/01/2012 | 1 | Other *Onchocerca* spp. |
| 34500 | BADARA KARABORO | 21/05/2012 | 1 | *Onchocerca volvulus* |
| 34501 | BADARA KARABORO | 21/05/2012 | 1 | *Onchocerca volvulus* |
| 34498 | BADARA KARABORO | 26/06/2012 | 1 | *Onchocerca volvulus* |
| 34499 | BADARA KARABORO | 26/06/2012 | 2 | *Onchocerca volvulus* |
| 34492 | BADARA KARABORO | 27/06/2012 | 2 | *Onchocerca volvulus* |
| 34493 | BADARA KARABORO | 27/06/2012 | 1 | *Onchocerca volvulus* |
| 34496 | BADARA KARABORO | 27/06/2012 | 1 | *Onchocerca volvulus* |
| 34497 | BADARA KARABORO | 27/06/2012 | 1 | *Onchocerca volvulus* |
| 34595 | BADARA KARABORO | 29/07/2012 | 1 | *Onchocerca volvulus* |
| 34594 | BADARA KARABORO | 12/08/2012 | 1 | Other *Onchocerca* spp. |
| 34482 | BADARA KARABORO | 21/09/2012 | 4 | Other *Onchocerca* spp. |
| 34478 | BADARA KARABORO | 18/10/2012 | 2 | *Onchocerca volvulus* |
| 34479 | BADARA KARABORO | 18/10/2012 | 1 | *Onchocerca volvulus* |
| 34484 | BADARA KARABORO | 16/12/2012 | 2 | *Onchocerca volvulus* |
| 34540 | BODADIOUGOU | 20/01/2012 | 1 | Other *Onchocerca* spp. |
| 34541 | BODADIOUGOU | 20/01/2012 | 3 | Other *Onchocerca* spp. |
| 34545 | BODADIOUGOU | 20/01/2012 | 1 | *Onchocerca volvulus* |
| 34546 | BODADIOUGOU | 20/01/2012 | 1 | Other *Onchocerca* spp. |
| 34536 | BODADIOUGOU | 21/01/2012 | 2 | Other *Onchocerca* spp. |
| 34537 | BODADIOUGOU | 21/01/2012 | 2 | *Onchocerca volvulus* |
| 34544 | BODADIOUGOU | 21/01/2012 | 1 | *Other Onchocerca spp.* |
| 34533 | BODADIOUGOU | 17/02/2012 | 2 | *Onchocerca volvulus* |
| 34534 | BODADIOUGOU | 17/02/2012 | 1 | *Onchocerca volvulus* |
| 34535 | BODADIOUGOU | 17/02/2012 | 1 | Other *Onchocerca* spp. |
| 34524 | BODADIOUGOU | 17/02/2012 | 1 | Other *Onchocerca* spp. |
| 34521 | BODADIOUGOU | 17/02/2012 | 2 | Other *Onchocerca* spp. |
| 34528 | BODADIOUGOU | 17/02/2012 | 2 | Other *Onchocerca* spp. |
| 34530 | BODADIOUGOU | 18/02/2012 | 1 | Other *Onchocerca* spp. |
| 34531 | BODADIOUGOU | 18/02/2012 | 1 | Other *Onchocerca* spp. |
| 34532 | BODADIOUGOU | 18/02/2012 | 2 | Other *Onchocerca* spp. |
| 34529 | BODADIOUGOU | 26/03/2012 | 1 | Other *Onchocerca* spp. |
| 34487 | BODADIOUGOU | 26/03/2012 | 2 | Other *Onchocerca* spp. |
| 34523 | BODADIOUGOU | 26/03/2012 | 1 | *Onchocerca volvulus* |
| 34526 | BODADIOUGOU | 26/03/2012 | 2 | Other *Onchocerca* spp. |
| 34486 | BODADIOUGOU | 26/03/2012 | 1 | Other *Onchocerca* spp. |
| 34525 | BODADIOUGOU | 27/03/2012 | 1 | Other *Onchocerca* spp. |
| 34520 | BODADIOUGOU | 27/03/2012 | 12 | Other *Onchocerca* spp. |
| 34518 | BODADIOUGOU | 18/04/2012 | 1 | Other *Onchocerca* spp. |
| 34519 | BODADIOUGOU | 18/04/2012 | 1 | Other *Onchocerca* spp. |
| 34514 | BODADIOUGOU | 19/04/2012 | 1 | Other *Onchocerca* spp. |
| 34515 | BODADIOUGOU | 19/04/2012 | 1 | Other *Onchocerca* spp. |
| 34516 | BODADIOUGOU | 19/04/2012 | 4 | *Onchocerca volvulus* |
| 34517 | BODADIOUGOU | 19/04/2012 | 3 | Other *Onchocerca* spp. |
| 34509 | BODADIOUGOU | 16/05/2012 | 1 | Other *Onchocerca* spp. |
| 34510 | BODADIOUGOU | 16/05/2012 | 2 | Other *Onchocerca* spp. |
| 34511 | BODADIOUGOU | 16/05/2012 | 2 | *Onchocerca volvulus* |
| 34512 | BODADIOUGOU | 16/05/2012 | 1 | *Onchocerca volvulus* |
| 34513 | BODADIOUGOU | 16/05/2012 | 2 | Other *Onchocerca* spp. |
| 34505 | BODADIOUGOU | 17/05/2012 | 1 | *Onchocerca volvulus* |
| 34506 | BODADIOUGOU | 17/05/2012 | 1 | Other *Onchocerca* spp. |
| 34507 | BODADIOUGOU | 17/05/2012 | 1 | *Onchocerca volvulus* |
| 34508 | BODADIOUGOU | 17/05/2012 | 2 | Other *Onchocerca* spp. |
| 34503 | BODADIOUGOU | 09/06/2012 | 4 | *Onchocerca volvulus* |
| 34504 | BODADIOUGOU | 09/06/2012 | 1 | *Onchocerca volvulus* |
| 34502 | BODADIOUGOU | 10/06/2012 | 2 | *Onchocerca volvulus* |
| 34596 | BODADIOUGOU | 10/08/2012 | 1 | Other *Onchocerca* spp. |
| 34473 | BODADIOUGOU | 16/09/2012 | 2 | Other *Onchocerca* spp. |
| 34474 | BODADIOUGOU | 16/09/2012 | 1 | Other *Onchocerca* spp. |
| 34475 | BODADIOUGOU | 17/09/2012 | 1 | Other *Onchocerca* spp. |
| 34476 | BODADIOUGOU | 17/09/2012 | 3 | Other *Onchocerca* spp. |
| 34477 | BODADIOUGOU | 17/09/2012 | 1 | *Onchocerca volvulus* |
| 34480 | BODADIOUGOU | 17/10/2012 | 7 | *Onchocerca volvulus* |
| 34481 | BODADIOUGOU | 18/10/2012 | 1 | *Onchocerca volvulus* |
| 34485 | BODADIOUGOU | 14/12/2012 | 1 | *Onchocerca volvulus* |
| 34542 | BOLIBANA | 23/01/2012 | 3 | Other *Onchocerca* spp. |
| 34543 | BOLIBANA | 23/01/2012 | 1 | Other *Onchocerca* spp. |
| 34494 | BOLIBANA | 28/06/2012 | 1 | *Onchocerca volvulus* |
| 34495 | BOLIBANA | 28/06/2012 | 1 | *Onchocerca volvulus* |
| 34592 | BOLIBANA | 27/07/2012 | 1 | Other *Onchocerca* spp. |
| 34598 | BOLIBANA | 27/07/2012 | 1 | Other *Onchocerca* spp. |
| 34593 | BOLIBANA | 28/07/2012 | 8 | Other *Onchocerca* spp. |
| 34589 | BOLIBANA | 15/08/2012 | 1 | Other *Onchocerca* spp. |
| 34590 | BOLIBANA | 15/08/2012 | 1 | Other *Onchocerca* spp. |
| 34597 | BOLIBANA | 19/05/2012 | 1 | Other *Onchocerca* spp. |
| 34591 | BOLIBANA | 15/08/2012 | 1 | Other *Onchocerca* spp. |
| 34599 | BOLIBANA | 15/08/2012 | 1 | Other *Onchocerca* spp. |
